# RNA splicing in health and disease

**DOI:** 10.1186/s43556-026-00554-w

**Published:** 2026-08-21

**Authors:** Huining Huang, Yao Yu, Qian Zhou, Guangtong Deng, Yayun Li, Furong Zeng

**Affiliations:** 1 Department of Dermatology, Huiya Hospital of The First Affiliated Hospital, Sun Yat-Sen University, Huizhou, 516000 China; 2 Department of Dermatology, Xiangya Hospital, Central South University, Changsha, China; 3 National Engineering Research Center of Personalized Diagnostic and Therapeutic Technology, Changsha, China; 4 Furong Laboratory, Changsha, Hunan China; 5 Hunan Key Laboratory of Skin Cancer and Psoriasis, Hunan Engineering Research Center of Skin Health and Disease, Xiangya Hospital, Central South University, Changsha, China; 6 National Clinical Research Center for Geriatric Disorders, Xiangya Hospital, Central South University, Changsha, China; 7 Department of Dermatology, The Third Xiangya Hospital, Central South University, Changsha, Hunan 410013 China; 8 Department of Oncology, Xiangya Hospital, Central South University, 87 Xiangya Road, Changsha, Hunan China

**Keywords:** RNA splicing, Alternative splicing, Splicing regulation, Disease pathogenesis, Therapeutic targeting

## Abstract

RNA splicing expands the functional output of eukaryotic genomes by enabling individual precursor messenger RNA (pre-mRNA) to generate multiple mature transcripts with protein‑coding and regulatory properties. Its fidelity and plasticity depend on coordinated interactions among the spliceosome, trans-acting splicing factors, cis-regulatory elements, and chromatin- and RNA-associated regulatory mechanisms. However, how these components collectively generate cell- and tissue-specific splicing programs, and how their disruption drives disease, remain incompletely understood. In this review, we integrate the molecular regulation of RNA splicing with its physiological, pathological and therapeutic consequences. We describe how spliceosome assembly, splicing regulatory elements, splicing factors, epigenetic modifications, and post-transcriptional processes determine splice-site selection. We then examine how regulated isoform programs support development, tissue specialization, homeostasis, circadian timing, and stress adaptation, and how their failure contributes to cancer and diverse non-neoplastic diseases. In cancer, we highlight the bidirectional interplay between splicing dysregulation and the tumor microenvironment, through which metabolic reprogramming and immune suppression reinforce aberrant splicing. Finally, we assess strategies that modulate the spliceosome, splicing-factor activity, or disease-associated transcripts, and present a perspective on how multi-omics, artificial intelligence, targeted delivery, and combination with immunotherapy could collectively advance the discovery and precision of splicing-directed therapies. We suggest that safe clinical translation will require greater selectivity, reduced off-target toxicity, and preservation of essential physiological splicing.

## Introduction

RNA splicing is a fundamental, conserved post-transcriptional process that removes non-coding intronic sequences and ligates coding exons from precursor messenger RNA (pre-mRNA) to generate mature transcripts [1]. It includes constitutive splicing, which produces a single transcript in a fixed pattern, and alternative splicing, which enables one gene to generate multiple mRNA isoforms through differential splice-site selection. By expanding transcriptomic and proteomic diversity beyond the constraints of gene number, RNA splicing provides a key molecular basis for cellular plasticity and phenotypic complexity [1–3].

The fidelity and flexibility of splicing are maintained by the spliceosome and splicing factors (SFs) [4]. The spliceosome is a highly dynamic ribonucleoprotein complex that recognizes splice sites and catalyses the two-step transesterification reactions required for intron removal and exon ligation [5, 6]. Together with cis-regulatory elements and trans-acting SFs, this machinery executes the splicing code and enables transcriptomes to respond to developmental cues and environmental stimulation [3, 7–10]. In addition, epigenetic regulators, including DNA methylation, N6‑methyladenosine modification, N7‑methylguanosine modification, RNA editing, histone modifications, non-coding RNAs (ncRNAs), higher-order chromatin conformation, and nucleosome positioning, provide multilayered and context-specific control over splicing decisions [11–15].

However, the complexity that enables precise splicing regulation also renders this system vulnerable to perturbation. Genetic mutations, environmental stressors such as hypoxia and oxidative stress, and aberrant intracellular signalling can disrupt spliceosome components or SF activity, leading to exon skipping, intron retention, cryptic splice-site activation, and isoform imbalance [3]. These abnormalities are widely implicated in cancer and in non-neoplastic diseases affecting the nervous, cardiovascular, respiratory, digestive, and other systems [16–19]. As mechanistic understanding has deepened, RNA splicing has also emerged as a therapeutically tractable process, with small-molecule modulators, kinase inhibitors, splice-switching antisense oligonucleotides (ASOs), trans-splicing-mediated RNA exon editing, and CRISPR-based RNA-targeting tools expanding the landscape of splicing-targeted medicine [20–26].

In this review, we provide an integrated overview of RNA splicing in health and disease. We first summarize the molecular mechanisms of spliceosome assembly and alternative splicing, then discuss the regulatory networks that shape splicing decisions. We further examine how alternative splicing supports physiological complexity across development, tissue specialization, homeostasis, circadian timing, and stress adaptation, before considering how disruption of these programs contributes to oncological and non-oncological diseases. Finally, we summarize current and emerging therapeutic strategies targeting RNA splicing, and highlight key unresolved challenges and future directions for the field.

## Molecular mechanisms of RNA splicing

RNA splicing is a central post-transcriptional process that removes introns from pre-mRNA and ligates exons to generate mature transcripts [5]. Beyond intron removal, RNA splicing constitutes a fundamental regulatory layer of gene expression. Through alternative splice-site selection, a single gene can generate multiple transcript isoforms, markedly expanding proteomic diversity and functional complexity [19].

**Fig. 1:**
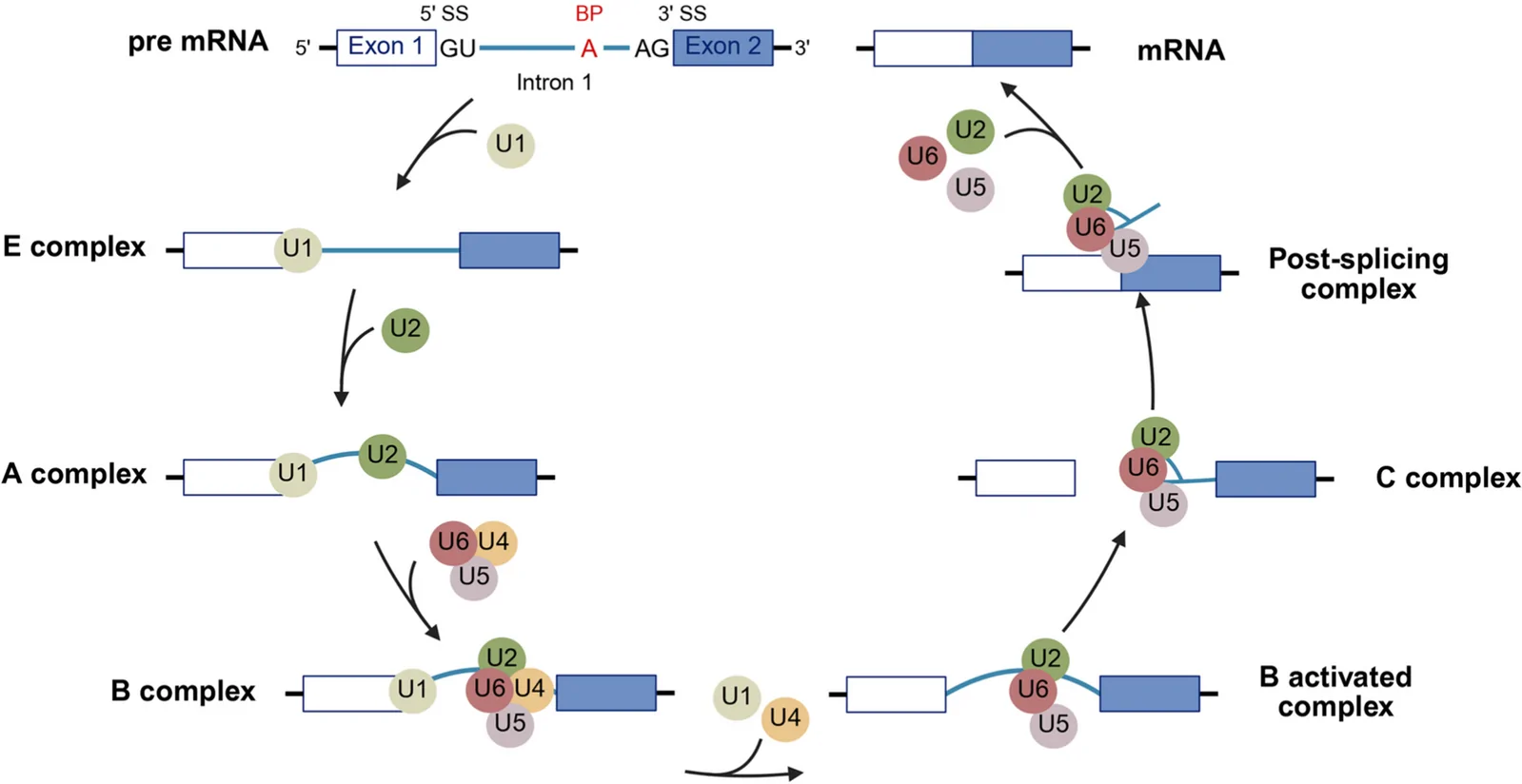
The process of RNA splicing. U1 snRNP binds to the 5’ splice site (5’ SS), and begins to form the E complex. U2 snRNP binds to the BP, begins to form the A complex, and brings the exons at both ends closer together. The U4/U6.U5 tri-snRNP complex is recruited and bound to the pre-mRNA to form the B complex. With the release of U1 and U4, the B complex is activated and catalyzes two transesterification reactions. The first transesterification reaction: 5’ SS overlaps with BP and cuts the RNA strand to form a lasso structure, which becomes the C complex. Second transesterification: the first exon cleavage attacks the 3’ splice site (3’ SS), allowing the exons to interconnect to form mature mRNA as a post-splicing complex. The spliceosome then disintegrates, releasing U2/U5/U6. The two exons complete conjugation to form the final mature mRNA. Abbreviations: 3’ SS, 3’ splice site; 5’ SS, 5’ splice site; BP, branch point; mRNA, messenger RNA; pre-mRNA, precursor messenger RNA; U1, U1 small nuclear ribonucleoprotein; U2, U2 small nuclear ribonucleoprotein; U4, U4 small nuclear ribonucleoprotein; U5, U5 small nuclear ribonucleoprotein; U6, U6 small nuclear ribonucleoprotein

RNA splicing is catalyzed by the spliceosome, a highly dynamic ribonucleoprotein (RNP) machinery composed of five small nuclear ribonucleoproteins (U1, U2, and the U4/U6.U5 tri-snRNP) together with numerous conserved proteinaceous co-factors [5, 27]. Spliceosome assembly proceeds in a stepwise and tightly coordinated manner (Fig. 1), beginning with U1 snRNP recognition of the 5′ splice site (SS) of the pre-mRNA via complementary base pairing [27]. Simultaneously, the U2AF65 protein binds to the polypyrimidine tract (PPT) and interacts with the branch point binding protein (SF1), facilitating its anchoring to the branch point (BP), while U2AF35 binds to the 3’ SS. The assembly of these components constitutes the inactive E complex. Subsequently, mediated by U2AF, the U2 snRNP displaces SF1 to bind the BP through base pairing, transitioning the assembly into the A complex—a step rigorously regulated by numerous SFs. Upon stabilization of the A complex, the U4/U6/U5 tri-snRNP joins the assembly, triggering conformational rearrangements that bring the three critical splice sites into proximity. Within this tri-snRNP, U4 and U6 are tightly associated through RNA complementarity, whereas U5 is loosely tethered via protein–protein interactions, forming the pre-catalytic B complex [4, 5, 28].

Catalytic activation requires further structural remodeling. U1 snRNP dissociates, releasing the 5’ SS to be subsequently bound by U6 snRNP; simultaneously, the release of U4 snRNP liberates U6 small nuclear RNA (snRNA), allowing it to interact with U2 snRNA via base pairing. This restructuring ultimately generates the catalytically active B complex. Once the catalytic center is established, the spliceosome orchestrates two successive transesterification reactions. In the first step, the 5’ SS is cleaved and covalently linked to the branch point, forming a lariat structure as the assembly transitions into the C complex. This is immediately followed by the second transesterification reaction, where the liberated 5’ exon attacks the 3’ SS, resulting in the ligation of the two exons to form a mature mRNA. The intron is excised as a lariat and subsequently degraded. Following the reaction, the involved snRNP particles are released and recycled for subsequent rounds of spliceosome assembly and catalysis. Through this precise orchestration, introns are accurately removed, and exons are orderly ligated to produce functional, mature mRNA transcripts [5].

Functionally, RNA splicing can be broadly classified into constitutive splicing and alternative splicing [29, 30]. Constitutive splicing removes all introns and joins exons in their genomic order, producing a single dominant transcript [5, 30, 31]. By contrast, alternative splicing permits regulated selection among competing splice sites, generating distinct mRNA isoforms from the same pre-mRNA. Major patterns include exon skipping, mutually exclusive exons, intron retention (IR), and alternative 5’ or 3’ splice-site selection. Through these mechanisms, alternative splicing empowers organisms to produce a vast repertoire of functionally specialized proteins from a limited genome, facilitating adaptation to intricate physiological demands [30].

## Regulation of RNA splicing

RNA splicing is a tightly regulated post-transcriptional process that shapes transcriptome complexity and drives proteomic diversity in eukaryotes. Beyond the spliceosome machinery and core splice sites, splice-site selection is further regulated by two major layers of control: cis-regulatory elements together with their trans-acting factors, as well as epigenetic regulation mechanisms, including DNA methylation, RNA modifications such as N6-methyladenosine (m6A) modification, N7-methylguanosine (m7G) modification, RNA editing, histone modifications, ncRNAs, higher-order chromatin conformation, and nucleosome positioning.

### Cis-regulatory elements and trans-acting factors in the regulation of mRNA splicing

Splice-site selection in multi-exon pre-mRNAs is orchestrated by splicing regulatory elements (SREs)—short, conserved RNA sequences embedded in transcripts and recognized by specific RNA-binding proteins [27, 32]. Based on their location and function, SREs are classified into four categories: exonic splicing enhancers (ESEs), intronic splicing enhancers (ISEs), exonic splicing silencers (ESSs), or intronic splicing silencers (ISSs). Generally, enhancers are recognized by trans-acting factors, most notably the serine/arginine-rich (SR) protein family, facilitating splice-site recognition and exon inclusion [33]. Conversely, silencers interact with other types of trans-acting factors, such as hnRNPs, to restrict splice-site recognition and promote exon skipping [5, 34].

Trans-acting factors are proteins that interact with cis-regulatory elements to modulate gene expression. They bind to these elements within the pre-mRNA, forming complexes that influence spliceosome assembly, thereby achieving highly specific splice-site selection. Key families of trans-acting factors include the SR proteins and hnRNPs [35]. Additionally, other specific SFs, such as ESRP1/2 and MBNL1/2/3, bind to pre-mRNA to regulate spliceosome assembly and the efficiency of splice-site recognition [32].

### Epigenetic regulation of RNA splicing

**Fig. 2:**
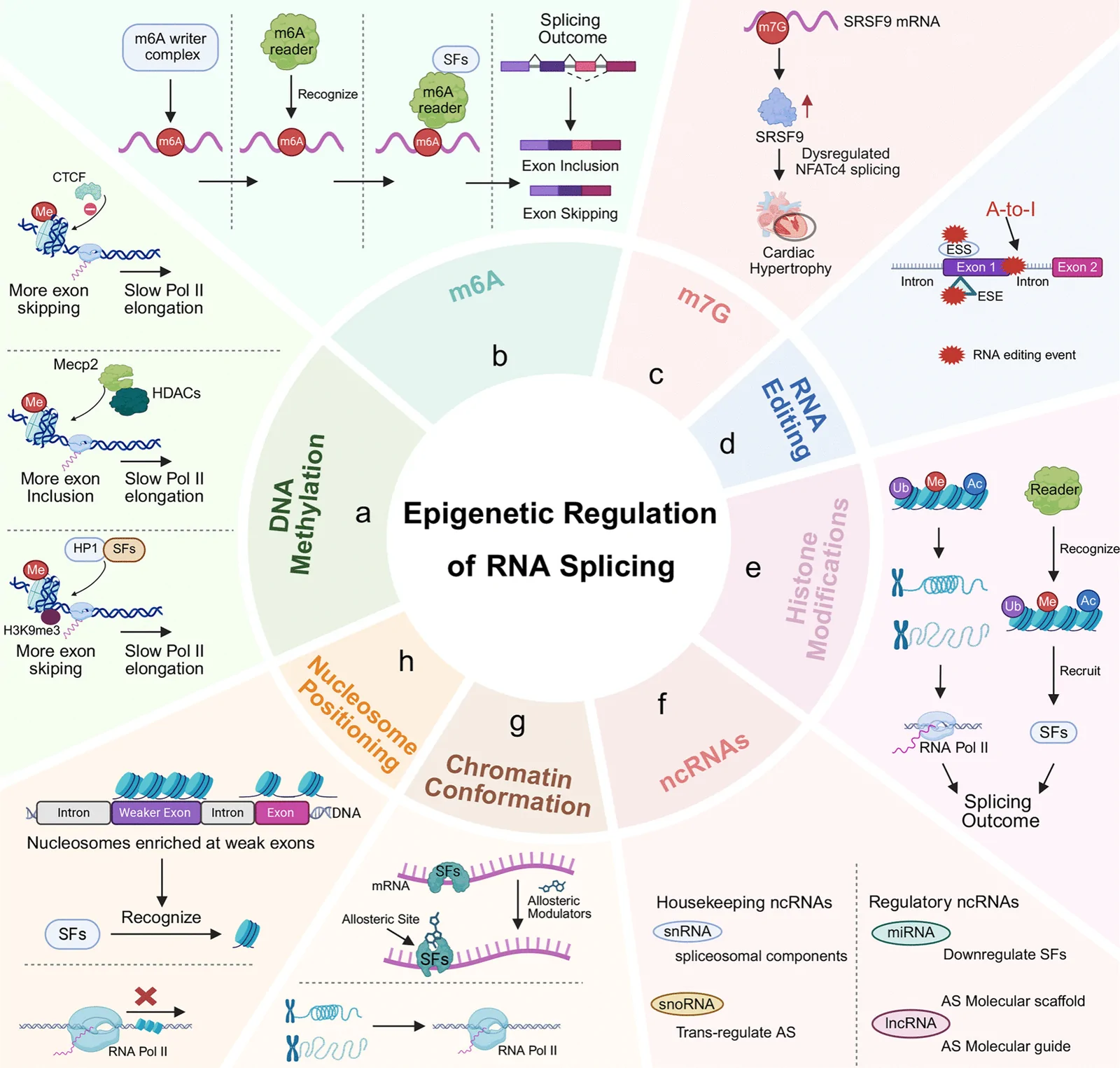
Epigenetic regulation of RNA splicing. **a** The mechanisms by which DNA methylation influences alternative splicing. DNA methylation inhibits CTCF binding; this releases a physical barrier, slowing Pol II elongation; MeCP2 binds to the methylated sequences and recruits HDAC enzymes, thereby slowing Pol II elongation; DNA methylation is frequently co-localized with histone modifications such as H3K9 methylation. The HP1 protein recognizes H3K9 methylation and recruits splicing factors, thereby slowing Pol II elongation. **b** The m6A writer complex catalyzes m6A modification on pre-mRNA. m6A reader proteins recognize modified sites and recruit splicing factors, thereby determining splicing outcomes. **c** m7G modification of SRSF9 mRNA upregulates SRSF9 expression, which promotes alternative splicing and stabilization of NFATc4, driving the hypertrophic phenotype. **d** RNA editing regulates splicing either by directly creating or disrupting canonical splice sites, or through SREs, such as ESEs or ESSs. **e** Histone modifications (including methylation, acetylation, and ubiquitination) regulate pre-mRNA splicing by altering chromatin conformation to influence the elongation rate of RNA polymerase II or recruiting specific transcriptional and splicing regulators. **f** Classification of major non-coding RNAs and their functional mechanisms in splicing. **g** Changes in chromatin conformation regulate splicing through both direct and indirect mechanisms. Direct: Certain chromatin remodeling complexes recruit snRNPs to participate in the assembly of core spliceosomal components. Indirect: Chromatin conformational changes alter the elongation rate of RNA polymerase II. **h** Nucleosome positioning regulates alternative splicing via two mechanisms: Nucleosomes mark weak exons and are recognized by SFs; Nucleosomes directly impede Pol II elongation to regulate splicing. Abbreviations: A-to-I, adenosine-to-inosine RNA editing; Ac, acetylation; AS, alternative splicing; CTCF, CCCTC-binding factor; DNA, deoxyribonucleic acid; ESE, exonic splicing enhancer; ESS, exonic splicing silencer; H3K9me3, trimethylation of lysine 9 on histone H3; HDACs, histone deacetylases; HP1, heterochromatin protein 1; lncRNA, long non-coding RNA; m6A, N6-methyladenosine; m7G, N7-methylguanosine; Me, methylation; MeCP2, methyl-CpG-binding protein 2; miRNA, microRNA; mRNA, messenger RNA; ncRNAs, non-coding RNAs; NFATC4, nuclear factor of activated T cells 4; RNA, ribonucleic acid; RNA Pol II, RNA polymerase II; SFs, splicing factors; snoRNA, small nucleolar RNA; snRNA, small nuclear RNA; SRSF9, serine/arginine-rich splicing factor 9; Ub, ubiquitination

Pre-mRNA splicing is a sophisticated process governed by diverse epigenetic mechanisms, including DNA methylation, m6A modification, m7G modification, RNA editing, histone modifications, ncRNAs, higher-order chromatin conformation, and nucleosome positioning. These processes collectively modulate the selection of alternative splice sites and the generation of transcript diversity (Fig. 2).

#### DNA methylation in splicing regulation

DNA methylation is a covalent epigenetic modification catalyzed by DNA methyltransferases (DNMTs), utilizing S-adenosylmethionine as a methyl donor to attach a methyl group to specific bases within the DNA sequence. This process regulates pre-mRNA splicing not only by modulating the elongation rate of RNA polymerase II (Pol II), which influences the recognition of splice signals, but also by altering histone modifications that facilitate the recruitment of splicing regulators. CCCTC-binding factor (CTCF), a DNA-binding protein with high sequence specificity, can act as a physical barrier to decelerate Pol II progression [36, 37]. This reduction in kinetic speed increases the selective efficiency of the spliceosome for weak splice signals, thereby promoting the inclusion of specific alternative exons. Beyond kinetic regulation, DNA methylation directly modulates splicing complex assembly through crosstalk with histone modifications. Genomic regions with high DNA methylation levels frequently co-localize with the repressive histone mark histone H3 lysine 9 trimethylation (H3K9me3). Heterochromatin Protein 1 (HP1) specifically recognizes and binds H3K9me3-modified histones, and in turn recruits the core spliceosomal component U2 snRNP to the actively transcribed genomic locus. This axis positions HP1 as a molecular bridge that transduces DNA methylation signals directly to the splicing machinery (Fig. 2a) [15, 38].

#### N6-methyladenosine modification in splicing regulation

m6A is the most prevalent internal modification of mRNA in eukaryotes. The m6A landscape is dynamically governed by methyltransferases, termed "writers" (e.g., METTL3, METTL14, WTAP, and RBM15), and reversed by demethylases, known as "erasers" (e.g., FTO and ALKBH5) [39, 40]. The regulatory impact of m6A on alternative splicing is mediated by m6A-binding proteins, or "Readers," such as YTHDF1 and eIF3. The core methyltransferase complex—comprised of METTL3, METTL14, and WTAP (the WMM complex)—catalyzes m6A modification on pre-mRNAs to regulate splicing. Functional enrichment analysis by Ping et al. demonstrated that depletion of the WMM complex leads to a marked reduction in differentially expressed genes associated with RNA processing, underscoring the essential role of m6A writers in shaping transcript diversity through alternative splicing [41]. As a reader protein, YTHDF1 recognizes m6A-modified bases to activate downstream regulatory pathways. YTHDF1 identifies m6A sites within coding sequences and 3’-UTRs and recruits splicing regulators, such as members of the SRSF family [42]. Notably, SRSF3 and SRSF10 exhibit competitive binding for YTHDF1-associated regions, with SRSF3 exhibiting higher binding affinity. Under normal physiological conditions, YTHDF1 recognizes m6A and preferentially recruits SRSF3, thereby promoting exon retention (Fig. 2b) [42].

#### N7-methylguanosine modification in splicing regulation

m7G modification refers to the methylation occurring at the seventh nitrogen (N7) atom of the guanine base in RNA (Fig. 2c). Present across transfer RNA (tRNA), microRNA (miRNA), and internal mRNA sequences, m7G modification has emerged as a pivotal regulator of alternative splicing. The regulatory mechanisms of m7G on alternative splicing are multifaceted and operate at multiple levels [43]. On one hand, specific m7G methyltransferases, such as METTL1, can methylate the mRNA of key SFs (e.g., SRSF9) [44]. This internal modification upregulates their expression levels, thereby reshaping the alternative splicing patterns of downstream target genes. For instance, in models of cardiac hypertrophy, METTL1 induces m7G modification of SRSF9 mRNA, which subsequently promotes the alternative splicing and stabilization of NFATc4, thereby driving the hypertrophic phenotype. On the other hand, certain viral proteins, such as NSP14 from SARS-CoV-2, can introduce aberrant internal m7G modifications into host mRNAs. This activity directly interferes with canonical splicing processes, leading to increased IR and the activation of cryptic splice sites. Consequently, this epigenetic interference reprograms host gene expression to facilitate viral replication and survival [45].

#### RNA editing in splicing regulation

RNA editing refers to the post-transcriptional modification of primary transcripts through the insertion, deletion, or substitution of nucleotides, serving as a critical bridge between genomic encoding and proteomic diversity. Within the regulatory landscape of splicing, RNA editing precisely intervenes in splice-site selection by physically altering splicing signals or modulating the recruitment of regulatory factors [27, 46]. This interplay primarily manifests through two distinct mechanisms (Fig. 2d).

RNA editing can remodel transcript architecture by directly creating or disrupting canonical splice sites. A quintessential example is the auto-regulatory feedback loop of Adenosine Deaminase Acting on RNA 2 (ADAR2). ADAR2 catalyzes the deamination of adenosine (A) to inosine (I) within its own pre-mRNA. Since the splicing machinery interprets inosine as guanosine (G), the conversion of an AA dinucleotide to AI creates a functional 3’ splice acceptor site (AG). This editing-induced splicing activation leads to the inclusion of a 47-nucleotide cassette, resulting in a frame-shift mutation that yields a non-functional protein isoform. This mechanism maintains cellular ADAR2 homeostasis. Beyond auto-regulation, similar A-to-I editing within the 5-HT2CR (serotonin receptor 2 C) exonic regions directly shifts its alternative splicing patterns, altering receptor signaling efficacy [47, 48].

RNA editing indirectly influences splicing by creating or disrupting SREs, thereby modulating the binding dynamics of trans-acting splicing regulators to target transcripts. SREs, such as ESEs or ESSs, are highly sequence-sensitive. Research by Szabo et al. demonstrated that A-to-I editing within these motifs can significantly shift the binding affinity of core SFs for these sequences [49]. For instance, an editing event may activate a cryptic enhancer, recruiting SR proteins to promote exon inclusion, or conversely, abolish a silencer motif to prevent the binding of hnRNPs (e.g., hnRNP A1), thereby suppressing exon skipping or IR [50]. This layer of regulation is particularly prevalent in the central nervous system; for example, in the glutamate receptor GABRA3, RNA editing reshapes the protein-RNA interaction network to ensure the precise spatiotemporal expression of specific isoforms during neural development [51].

#### Histone modifications in splicing regulation

Histones are core DNA-binding proteins that form the fundamental structural unit of eukaryotic chromatin. Their N-terminal tails and globular domains contain a wide array of amino acid residues that are subject to histone post-translational modifications (hPTMs), including methylation, acetylation, and ubiquitination. A large body of robust evidence has established that these modifications govern pre-mRNA splicing through two primary mechanisms: altering chromatin compaction and configuration, or serving as docking sites to recruit specific transcriptional and splicing regulators (Fig. 2e) [15, 52].

A prominent example of this regulation is observed in Fibroblast Growth Factor Receptor 2 (FGFR2), which generates two mutually exclusive, tissue-specific isoforms, FGFR2-IIIb and FGFR2-IIIc- via tightly regulated alternative splicing. Hu et al. demonstrated that in mesenchymal cells, the enrichment of H3K36me3 and H3K4me1 near FGFR2 splice sites influences the recruitment of SFs to drive preferential production of the FGFR2-IIIc isoform. Conversely, the local chromatin landscape at this locus is marked predominantly by H3K27me3 and H3K4me3, which promotes the retention of exon IIIb to generate the FGFR2-IIIb isoform [53, 54].

The functional significance of these marks was further validated by Luco et al., who showed that manipulating H3K36me3 or H3K4me3 levels—via the overexpression or knockdown of specific histone methyltransferases—could effectively reprogram FGFR2 splicing patterns [53]. These findings provide definitive evidence for the direct role of histone modifications in orchestrating alternative splicing. Furthermore, in 2012, Enroth et al. identified that histone modifications are decisive factors in determining whether an exon is included or excluded during exon skipping events, reinforcing the existence of a conserved “chromatin-splicing” regulatory axis [55].

#### Non-coding RNAs in splicing regulation

ncRNAs are transcripts derived from the genome that do not possess protein-coding capabilities. Based on their biological functions, ncRNAs can be categorized into housekeeping ncRNAs and regulatory ncRNAs. Regulatory ncRNAs are further divided into short regulatory ncRNAs (such as small interfering RNA (siRNA), miRNA, and PIWI-interacting RNA (piRNA)) and long regulatory ncRNAs (such as long non-coding RNA (lncRNA)). Beyond their established roles in post-transcriptional gene regulation, ncRNAs modulate pre-mRNA splicing through both direct and indirect mechanisms, including crosstalk with histone modification pathways [55].

Among the diverse family of housekeeping RNAs, the regulation of splicing is primarily mediated by snRNAs. snRNAs, such as U1 and U2, function as the core catalytic components of the spliceosome [56, 57]. They directly recognize and define splice sites via complementary base pairing with consensus sequences in pre-mRNA, and drive the two-step transesterification reactions that are central to the splicing process. In addition, certain housekeeping small nucleolar RNAs (snoRNAs), such as SNORD115, have been demonstrated to participate in trans-regulation of alternative splicing, exemplified by their role in modulating exon selection in genes like the serotonin receptor 2 C (Htr2c) [58].

Within the class of short regulatory ncRNAs, specific miRNAs exert indirect, cross-pathway regulation of splicing by downregulating the expression of key SFs, thereby reshaping the splicing profiles of downstream target genes [59, 60]. LncRNAs exhibit the most diverse mechanisms in splicing regulation. Some lncRNAs interact directly with SFs and modulate their post-translational modifications, thereby altering their activity and indirectly shaping global splicing profiles [61]. In the context of SRPK1-mediated signaling, for example, the expression of lncRNAs such as MALAT1 and TUG1 fluctuates dynamically in response to SRPK1 activity, implicating these transcripts in kinase-regulated splicing networks [61].

Furthermore, certain lncRNAs serve as molecular scaffolds [62]. In hepatocellular carcinoma, the highly expressed lncRNA MALAT1 simultaneously binds two SFs, PTBP1 and PSF, stabilizing their interaction and anchoring the complex to chromatin-associated nascent RNA [63, 64]. This scaffolding function enables coordinated, locus-specific regulation of alternative splicing in downstream target genes. Additionally, many lncRNAs act as molecular guides to direct SFs to specific pre-mRNA targets. In colorectal cancer, the lncRNA MIR99AHG specifically recruits PTBP1 to SMARCA1 transcripts [65]. By modulating the binding site preference of PTBP1, it promotes exon inclusion of a specific exon, leading to the generation of an oncogenic isoform. Similarly, the neuron-specific lncRNA Gomafu regulates the splicing of key genes involved in neurodevelopment, such as DISC1 and ERBB4, by recruiting SFs like SF1 to their pre-mRNA targets. Downregulation of Gomafu disrupts the precise recruitment of these factors, resulting in aberrant splicing patterns that contribute to neuronal dysfunction (Fig. 2f) [66].

#### Chromatin conformation in splicing regulation

Chromatin is the primary carrier of genomic information, composed of DNA and a diverse repertoire of histone and non-histone proteins whose combinatorial interactions define its structural and functional heterogeneity (Fig. 2g) [67]. Both histone and non-histone proteins that constitute chromatin can directly influence the assembly of the spliceosome through protein–protein interactions, thereby dictating alternative splicing outcomes [15]. Kornblihtt and colleagues demonstrated that depolarization of neuronal cells can trigger the skipping of exon 18 during the splicing of the Neural Cell Adhesion Molecule (NCAM) [68]. This skipping may be related to an increase in the elongation rate of Pol II, which results from the loosening of chromatin structure after depolarization. Recently, the same team found that using siRNA to target sequences flanking alternative exons can alter splicing outcomes. They proposed that this regulatory mechanism is similar to the principle of transcriptional gene silencing by siRNA interference, both being achieved by promoting heterochromatin formation, which in turn impedes the elongation of Pol II [69]. This evidence demonstrates that the regulation of splicing by chromatin structure can be realized through a kinetic model affecting transcription-coupled processes. The research team led by Batsché found that the Brm protein, a member of the SWI/SNF family of chromatin remodeling complexes, can recruit U1 snRNPs and U5 snRNPs to participate in spliceosome assembly [70]. Brm also interacts with the RNA-binding protein Sam68, promoting its binding to the alternative exon of CD44, ultimately leading to the inclusion of this exon. Furthermore, the Brm protein can accumulate Pol II in the region of the alternative exon, thereby reducing the elongation rate of Pol II. Thus, it is evident that changes in chromatin conformation can directly regulate alternative splicing, and can also indirectly influence it by altering the elongation rate of Pol II [70]. These two regulatory modes are not mutually exclusive but rather coexist and crosstalk to fine-tune splicing precision.

#### Nucleosome positioning in splicing regulation

The nucleosome, composed of the core histone proteins H2A, H2B, H3, H4 and approximately 147 bp of DNA, is the fundamental repeating unit of eukaryotic chromatin [71]. Substantial evidence indicates that nucleosome positioning is intimately linked to alternative splicing. ChIP-seq analyses have revealed that in the genomes of humans (Homo sapiens L.), fruit flies (Drosophila melanogaster M.), nematodes (Caenorhabditis elegans M.), and Japanese medaka (Oryzias latipes T.), the distribution density of nucleosomes is significantly higher in exon regions compared to intron regions [72]. Furthermore, nucleosome density within exons correlates positively with the length of flanking intron sequences. Pseudo-exons located between two strong splice sites lack nucleosome distribution. Additionally, the nucleosome density surrounding exons that are included is greater than that around exons that are skipped [15]. These findings suggest that nucleosome positioning not only serves as an epigenetic marker to distinguish exons from introns but also participate in the regulation of splicing (Fig. 2h). The potential regulatory roles of nucleosome positioning in RNA splicing can be summarized in two key mechanisms: (1) Nucleosome enrichment at weak exons functions as a molecular marker; specific SFs can recognize nucleosomes and interact with other spliceosome components to facilitate spliceosome assembly and modulate splicing outcomes [73]. (2) Nucleosomes can alter the elongation rate of Pol II, hindering its progression and thus regulating RNA splicing [74].

## Role of RNA splicing in normal physiological functions

Alternative splicing markedly expands proteomic diversity and provides a fundamental molecular substrate for the phenotypic complexity of higher eukaryotes. The relationship between genome size and organismal complexity is not linear; instead, regulated RNA processing substantially amplifies coding and functional output from a relatively limited number of genes [30]. Through modular recombination of a single pre-mRNA, splicing challenges the traditional “one gene, one polypeptide” paradigm and enables approximately 20,000 protein-coding genes in the human genome to generate extensive repertoires of isoforms with distinct biochemical properties, subcellular localizations and interaction networks [75]. This expanded isoform landscape constitutes a central regulatory layer that underpins tissue specialization, developmental transitions and physiological adaptability.

### Orchestrating developmental programming and cell fate commitment

Alternative splicing establishes developmental trajectories through temporally coordinated isoform transitions that guide organogenesis and lineage specification, aligning cellular properties with stage-specific physiological demands.

Tissue-enriched SFs, including members of the MBNL, CELF and RBM families, exhibit dynamic and coordinated expression patterns across ontogeny, driving widespread transitions from embryonic to adult isoforms [76]. These transitions ensure that structural and signaling modules are precisely adapted to postnatal physiological requirements. Another notable example is found in the myocardium, where the titin isoform, regulated by RBM20, transitions from a highly elastic fetal variant to a more rigid adult variant, thereby adapting to the significant increase in cardiac workload after birth [77]. Developmental alternative splicing also participates directly in cell fate commitment by reshaping the functional domains of transcriptional regulators. PTBP1 modulates neuronal differentiation through regulation of a developmental splicing switch in DPF2 [78]. Similarly, in skeletal muscle developmental biology, studies have demonstrated that YBX1 governs the fate of BMSCs (bone marrow mesenchymal stromal cells) through the fine-tuned control of RNA splicing [79].

### Refining cellular identity and function in mature tissues

Following developmental establishment, alternative splicing sustains and refines cellular identity within mature tissues. Rather than redefining lineage fate, splicing at this stage stabilizes cell-type-specific isoform repertoires that confer functional precision and regional specialization.

In the auditory system, alternative splicing of potassium calcium-activated channel subfamily M alpha 1 (KCNMA1) in inner ear hair cells generates potassium channel isoforms with distinct kinetic properties and calcium sensitivities [80]. The graded distribution of these isoforms along the cochlea underlies frequency discrimination and auditory fine-tuning [81]. In the immune system, CD44 produces hundreds of isoforms through combinatorial inclusion of ten variable exons, diversifying adhesion, migration, and activation programs in lymphocytes [82, 83]. This context-dependent isoform repertoire enables immune cells to dynamically adjust receptor properties in response to inflammatory cues or lymphoid homing signals. In the nervous system, the neurexin genes (NRXN1, NRXN2 and NRXN3) undergo extensive alternative splicing to generate thousands of protein isoforms. This diversity enables them to bind to various postsynaptic ligands, allowing tens of thousands of synapses to connect precisely to their correct target cells [84].

### Maintaining homeostasis and circadian rhythm

Alternative splicing sustains physiological homeostasis and coordinates circadian rhythmicity across mammalian tissues and organ systems [85]. Acting through a complex regulatory network of SFs, it stabilizes cellular steady states via negative feedback loops, while enabling state transitions through positive feedback circuits. This splicing-dependent regulation is essential for homeostatic control across multiple core physiological domains, including glucose and lipid metabolism, blood pressure stability, glycemic control, and immune homeostasis [85–90]. For instance, loss of the spliceosomal component Usp39 leads to aberrant alternative splicing of autophagy-related genes such as Hsf1, resulting in impaired autophagy and hepatic lipid accumulation, underscoring the critical role of Usp39-mediated splicing in preserving liver lipid homeostasis [91]. The endoplasmic reticulum protein Nogo-B interacts with proglucagon (proGCG), impeding its intracellular trafficking and PCSK1-mediated cleavage, thereby regulating GLP-1 biosynthesis and downstream insulin secretion to maintain glycemic homeostasis [92]. Splicing regulation is also tightly coupled to the core circadian clock system. Core clock-controlled genes employ mechanisms such as IR to dynamically modulate mRNA stability and translational efficiency, enabling precise calibration of circadian oscillation frequency at the molecular level [93]. The abundance of core clock genes Per2 and Per3 is dynamically regulated by IR. Increased IR levels reduce functional mature transcripts, thereby delaying the peak accumulation of Period proteins and significantly prolonging the circadian period. This directly links splicing-mediated control of mRNA stability to the tuning of circadian cycle length [94, 95].

### Mediating adaptive stress responses and organismal self-protection

Beyond maintaining basal physiological functions, alternative splicing also mediates adaptive stress responses and organismal self-protection. Acting as a highly efficient “immediate response element”, the splicing machinery mounts rapid reactions to diverse environmental stressors including hypoxia, heat shock, and DNA damage [96–98].

Under stress conditions, alternative splicing governs cell fate via isoform switching of apoptotic regulators, most notably the Bcl-x gene, where it precisely tunes the balance between the pro-apoptotic Bcl-xS and anti-apoptotic Bcl-xL isoforms [99]. Li et al. showed that UFD1s, a stress-responsive splice variant, is upregulated under stress to modulate IPMK ubiquitination. This regulates autophagy and fatty acid oxidation, preserving lipid homeostasis and attenuating progression of metabolic dysfunction-associated steatohepatitis (MASH) [100]. In the immune system, splicing-mediated self-protection operates through two complementary mechanisms. It promotes the switch from membrane-bound to secreted immunoglobulin isoforms during immune activation, and finely tunes the magnitude of immune responses by expanding the molecular diversity of key receptors such as CD45 [101].

## Aberrant RNA splicing and disease

The fidelity of RNA splicing is a fundamental prerequisite for maintaining physiological homeostasis in human tissues. A large body of robust evidence indicates that once this finely tuned regulatory mechanism collapses, aberrant splicing can drive the pathogenesis of a broad spectrum of human diseases by altering or abolishing the critical physiological functions of key proteins, with pathological involvement spanning all major systems [102, 103]. The following is a detailed overview categorized into two major dimensions: neoplastic diseases and non-neoplastic diseases.

### Aberrant RNA splicing in cancer

Aberrant RNA splicing is increasingly recognized as a core molecular feature of cancer rather than a passive consequence of malignant transformation. Alterations in spliceosomal components and SFs can reshape the transcriptome and influence multiple interconnected processes, including genome maintenance, epigenetic regulation, proliferation, apoptosis, angiogenesis, invasion, immune escape, metabolic adaptation, and therapeutic resistance. These effects are therefore best understood as a network-level process in which splicing dysregulation both drives and reinforces malignant evolution.

#### Genomic, epigenetic, and phenotypic consequences of cancer-associated RNA splicing

Aberrant RNA splicing can compromise genomic integrity by impairing DNA damage repair and altering R-loop homeostasis, thereby promoting replication stress [104, 105]. In myelodysplastic syndromes (MDS), telomere dysfunction represses the expression of the SF SRSF2, thereby disrupting the transcription of genes involved in DNA replication and repair, promoting genomic instability and pathological hematopoiesis [106]. Meanwhile, recurrent SF3B1 mutations in MDS reduce R-loop formation and abnormally accelerate replication-fork progression, resulting in replication stress [107]. In chronic lymphocytic leukemia (CLL) and breast cancer, SF3B1 mutations upregulate circATP9B, which promotes MYH9 degradation, disrupts the intranuclear actin network, and markedly suppresses homologous recombination repair of DNA double-strand breaks [108]. Persistent genome instability can progressively expand tumor clonal diversity and provide a permissive background for malignant progression.

Cancer-associated splicing also interacts extensively with epigenetic regulation. Across multiple tumor types, splicing abnormalities and epigenetic alterations frequently co-occur and cooperate to stabilize malignant transcriptional states [109–112]. Mutations or oncogenic activation of core SFs can directly alter the expression and function of epigenetic regulators. For example, oncogenic overactivation of SRSF6 in prostate cancer reprograms the alternative splicing of the histone chaperone HIRA, reducing the abundance of functional HIRA protein and generating dominant-negative truncated variants. These changes impair H3.3 histone deposition and dysregulate oncogenic AR and E2F pathways [113]. Splicing defects can also disrupt the integrity and chromatin targeting of remodeling complexes. In CLL and uveal melanoma, SF3B1 mutations induce aberrant BRD9 splicing, resulting in mislocalization of the non-canonical BAF complex at enhancer regions, altered chromatin accessibility, and upregulated MYC signaling [114, 115]. In addition, mutations in SFs can perturb DNA methylation homeostasis. SRSF2 mutations disrupt the correct splicing of transcripts encoding TET-family DNA hydroxylases, thereby impairing oxidative demethylation of 5-methylcytosine. SRSF2-dependent aberrant splicing of INTS3 can also promote its transcriptional silencing and is associated with promoter-specific hypermethylation at the INTS3 locus. The resulting disruption of local DNA methylation homeostasis may further enable tumor cells to remodel the immune microenvironment and facilitate immune escape [109].

At the phenotypic level, aberrant splicing directly influences multiple hallmarks of cancer [16]. In apoptosis regulation, imbalanced splicing of BCL2-family genes increases the BCL-xL/BCL-xS ratio and favors an anti-apoptotic state by suppressing the activity of BAX and BAK [116, 117]. Aberrant splicing of caspase-family genes can produce dominant-negative isoforms that interrupt apoptotic signaling cascades [118, 119], whereas MDM2 splice variants can enhance p53 degradation [120]. Together, these mechanisms contribute to apoptosis resistance in tumors such as glioblastoma and platinum-resistant ovarian cancer [121, 122]. Splicing dysregulation also sustains uncontrolled proliferation by altering the isoform composition of cell-cycle regulators. Dysregulated expression or mutation of SFs can modify the processing of key cell-cycle transcripts, generating functionally abnormal isoforms that disrupt normal checkpoint control [1]. In lung adenocarcinoma, for example, PUF60 overexpression promotes the retention of critical exons in CDC25C, thereby maintaining production of the full-length protein, facilitating G2/M checkpoint progression and accelerating tumor-cell proliferation [123]. Splicing-dependent regulation also contributes to tumor angiogenesis and vascular remodeling. SRPK1-mediated splicing imbalance alters the ratio between pro-angiogenic and anti-angiogenic VEGFA isoforms and, together with hyperactive truncated HIF-1α isoforms, promotes the transcription of angiogenic factors and tumor neovascularization [124]. Aberrant VE-cadherin splicing can additionally promote vasculogenic mimicry, generating non-endothelial perfusion routes that support rapid tumor growth and invasion in renal carcinoma [125]. Invasion and metastasis are likewise shaped by cancer-associated splicing programs. During local invasion, dysregulated SFs promote the production of pro-migratory isoforms such as MENA and CD44v, which remodel the cytoskeleton and facilitate epithelial–mesenchymal transition [126, 127]. During metastatic dissemination, an increased Bcl-xL/Bcl-xS ratio and FGFR2 exon-skipping events can enhance the resistance of circulating tumor cells to anoikis [99, 128]. Hotspot mutations in spliceosomal components such as SF3B1 and U2AF1, together with overexpression of SFs including SRSF1, have also been associated with increased risks of metastasis and recurrence across multiple solid tumors [4, 129].

#### Remodeling of the tumor immune microenvironment

Aberrant RNA splicing critically modulates the tumor immune microenvironment by disrupting antigen presentation, rewiring immune checkpoint signaling, inducing stromal immune polarity shifts, and triggering metabolic reprogramming [130]. For instance, recurrent mutations in SFs SF3B1 and SRSF2 cause exon skipping in tumor‑specific antigens such as NY‑ESO‑1 and MAGE‑A, generating truncated proteins lacking key epitopes; these cannot be properly processed or presented via MHC‑I molecules, enabling tumor evasion from CD8⁺ T‑cell immunosurveillance—a mechanism well‑validated in melanoma and CLL [131, 132]. Overexpression of the core SF SRSF1 can stabilize functional membrane-bound PD-L1 splice isoforms to sustain persistent T cell exhaustion. Meanwhile, it drives macrophage polarization toward the M2 phenotype and further constructs an immunosuppressive microenvironment, which serves as a vital mechanism underlying immune evasion in triple-negative breast cancer, hepatocellular cancer, and pancreatic cancer [133–135]. Additionally, aberrant splicing‑derived soluble CTLA‑4 isoforms (sCTLA‑4) competitively engage CD80/CD86 co‑stimulatory molecules and evade neutralization by clinical CTLA‑4 monoclonal antibodies, representing a hidden core mechanism underlying immune checkpoint inhibitor resistance [136].

Splicing dysregulation further drives tumor metabolic reprogramming, forming a bidirectional crosstalk and mutually reinforcing vicious cycle with immune suppression, which plays a pivotal role in tumor immune evasion. For example, specific aberrant RNA splicing in bladder cancer directly upregulates the glycolytic rate‑limiting enzyme PKM2, compelling tumor cells to adopt aerobic glycolysis in lieu of normal oxidative phosphorylation. This metabolic shift results in substantial lactate accumulation and progressive depletion of essential nutrients (e.g., glucose) within the microenvironment, which directly suppresses the proliferation and cytotoxic function of CD8⁺ T cells and NK cells, thereby profoundly crippling both specific and innate antitumor immunity; simultaneously, it induces massive infiltration and enrichment of regulatory T cells (Tregs), further dampening local immune responses [137]. Critically, this metabolic‑immune crosstalk is bidirectional: the lactate‑rich, nutrient‑deprived immunosuppressive milieu, in turn, potentiates aberrant SF activation, sustaining PKM2 overexpression and hyperactive glycolysis, which exacerbates microenvironmental dysregulation [138].

#### Aberrant splicing and therapy resistance

The same splicing programs that support tumor initiation and progression can also promote resistance across multiple treatment modalities. Aberrant splicing contributes to chemoresistance, targeted therapy resistance, immunotherapy resistance, and resistance to anti-angiogenic therapy, thereby facilitating treatment failure and disease recurrence [129].

Chemoresistance can arise through the combined effects of enhanced drug efflux, increased DNA damage repair, and suppression of apoptosis [139]. Under intermittent hypoxia, non-small-cell lung cancer cells reprogram the alternative splicing of C/EBP-β pre-mRNA towards the LAP isoform. LAP subsequently increases the expression of drug-efflux transporters, including ABCB1 and ABCC1, thereby promoting chemoresistance [140]. In lung adenocarcinoma, upregulation of SNRPA promotes retention of exon 8 in ERCC1, stabilizing the ERCC1–XPF DNA-repair complex and enhancing the removal of cisplatin-induced DNA adducts. This process directly contributes to resistance to platinum-based chemotherapy [141]. In osteosarcoma, ERCC6 mediates cisplatin resistance through two complementary mechanisms: enhancement of DNA damage repair and, in cooperation with HNRNPM, induction of aberrant BAX splicing together with activation of PI3K–AKT signaling, thereby suppressing apoptosis [142].

Resistance to targeted therapy can result from splicing-mediated remodeling of drug targets or activation of bypass signaling pathways [129]. Aberrant splicing of oncogenic drivers can alter protein structure or eliminate drug-binding domains while preserving oncogenic activity. It can also activate compensatory signaling pathways that bypass inhibition of the primary therapeutic target, thereby contributing to both intrinsic and acquired resistance [143, 144].

Immunotherapy resistance may result from the combined effects of reduced antigen presentation, altered immune-checkpoint isoforms, and an immunosuppressive tumor microenvironment. Aberrant splicing can simultaneously increase membrane-bound and soluble checkpoint molecules, while defects in antigen-processing and presentation pathways can reduce tumor immune visibility. Together, these alterations can promote primary or acquired resistance to immune-checkpoint blockade [129, 145, 146].

Splicing-dependent vascular remodeling may also influence responses to anti-angiogenic therapy [147]. Altered VEGFA isoform ratios can maintain pro-angiogenic signaling, whereas vasculogenic mimicry and compensatory angiogenic programs can bypass conventional anti-angiogenic targets and contribute to cross-resistance [148–150].

Taken together, aberrant RNA splicing is not merely a secondary consequence of tumor progression. Instead, it functions through an interconnected network that links genomic instability, epigenetic remodeling, malignant phenotypes, metabolic adaptation, and immune escape with therapeutic resistance [2, 4, 129, 151, 152]. These processes can mutually reinforce one another, stabilizing malignant cellular states and reducing responsiveness to multiple therapeutic modalities. This integrated framework provides a mechanistic basis for identifying splicing-associated vulnerabilities and developing strategies to overcome treatment resistance.

### Aberrant RNA splicing and non-neoplastic disease

**Fig. 3:**
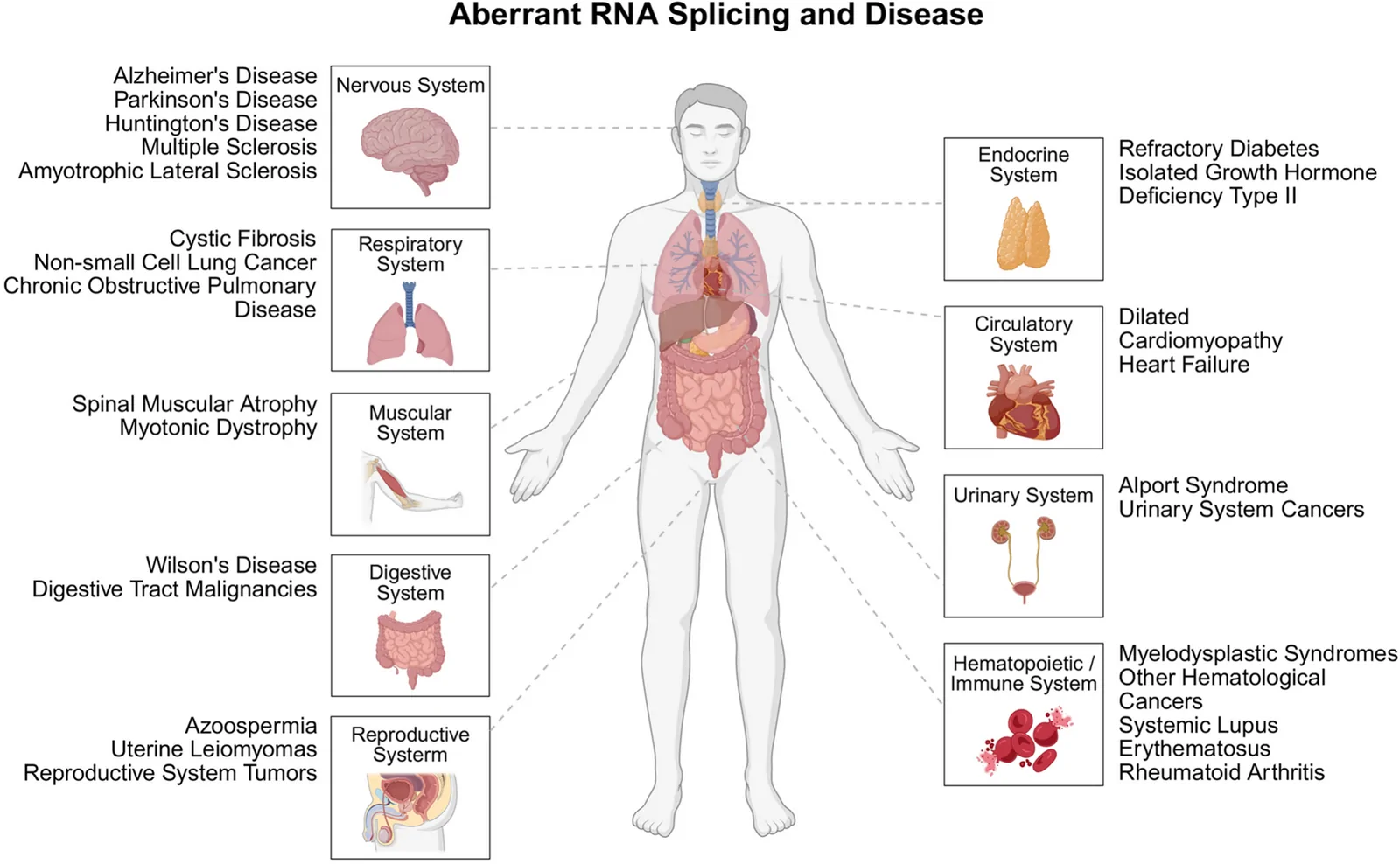
Summary of human diseases associated with aberrant RNA splicing. Aberrant RNA splicing serves as a fundamental pathogenic driver, disrupting proteomic integrity and cellular homeostasis. This figure delineates representative disorders across major physiological systems—ranging from neurodegenerative and muscular conditions to metabolic, immune, and malignant diseases. These clinical manifestations underscore the indispensable role of splicing fidelity in orchestrating organ-specific functions and systemic health

Beyond cancer, aberrant RNA splicing contributes to diverse non-neoplastic diseases through shared pathogenic mechanisms that manifest in a tissue-specific manner. These include loss of essential protein function, generation of dominant-negative or toxic isoforms, activation of cryptic splice sites, and disruption of cell-type-specific regulatory programs (Fig. 3, Table 1).

**Table 1:** Representative aberrant RNA splicing events and their pathogenic consequences across non-neoplastic diseases

System	Disease	Genes/Splicing Factors Involved	Main Consequence/Mechanism of Aberrant Splicing	Ref
**Nervous System**	Alzheimer’s disease	APP	Aberrant aggregation of U1 snRNP disrupts splicing of key genes like APP, accelerating amyloid plaque deposition	[153]
	Parkinson’s disease	LRRK2, etc	Dysregulation of alternative splicing of genes including LRRK2 is closely linked to the demise of dopaminergic neurons	[154]
	Huntington’s disease	HTT	Mis-splicing of the HTT gene pre-mRNA generates highly cytotoxic truncated protein fragments, directly inducing neuronal damage	[155]
	Multiple sclerosis	CELF1, NFAT5	Dysregulated expression of splicing factor CELF1 and its target gene NFAT5 observed in patient blood samples	[156]
	Amyotrophic lateral sclerosis	EAAT2	Altered alternative splicing of EAAT2 mRNA reduces glutamate transporter expression, impairing glutamate reuptake and leading to excitotoxic neuronal death	[157]
**Circulatory System**	Dilated cardiomyopathy	RBM20, TTN	RBM20 mutations lead to retention of overly compliant titin isoforms, impairing cardiac mechanical compliance	[158]
	Heart failure	RBM24, TP53, EIF4E	RBM24 binds p53 mRNA and interacts with EIF4E, blocking translation initiation complex assembly	[159]
**Respiratory System**	Cystic fibrosis	CFTR	Splicing defects lead to loss of CFTR protein function, causing mucociliary clearance dysfunction	[160–163]
	Chronic obstructive pulmonary disease	SRSF1, VEGF	Oxidative stress alters SRSF1, increasing inhibitory VEGF165b isoform ratio, inducing pulmonary microvascular endothelial cell apoptosis	[164]
**Hematopoietic and Immune Systems**	Myelodysplastic syndromes	SF3B1, etc	Mutations in core spliceosomal components generate aberrant transcripts with premature termination codons, blocking normal erythroid maturation	[165–167]
	Autoimmune diseases (systemic lupus erythematosus, rheumatoid arthritis)	CD45, FOXP3	Dysregulated splicing of immune receptors; imbalance of FOXP3 isoforms (FOXP3fl vs. FOXP3Δ2) impairs Treg suppressive function, disrupting immune homeostasis	[168, 169]
**Motor System**	Spinal muscular atrophy	SMN1, SMN2	SMN2 frequently undergoes exon 7 skipping, producing an unstable and dysfunctional SMN protein, resulting in skeletal muscle atrophy	[170, 171]
	Myotonic dystrophy	DMPK, MBNL1, CLCN1	Toxic RNA repeats sequester MBNL1, disrupting downstream splicing; mis-splicing of CLCN1 directly causes myotonia	[172, 173]
**Digestive System**	Wilson’s disease	ATP7B	Pathogenic mutations create cryptic splice sites, leading to mRNA with aberrant insertions or exon skipping, failing to produce functional ATP7B protein	[174, 175]
**Endocrine System**	Refractory diabetes	INSR	Mis-splicing of INSR exon 11 reduces its ligand affinity	[176]
	Isolated growth hormone deficiency type II	GH1	Aberrant skipping of GH1 exon 3 generates a dominant-negative 17.5 kDa growth hormone variant, impairing secretion of functional hormone	[177]
**Reproductive System**	Azoospermia	TEX14	TEX14 loss-of-function variants, including predicted splice-site-disrupting variants, impair intercellular bridge formation during meiosis, causing meiotic arrest and spermatogenic failure	[178]
**Urinary System**	Alport syndrome	COL4A5	Splice-site mutations (accounting for ~10–15% of X-linked cases)	[179]

*Abbreviations*: *APP* amyloid precursor protein, *ATP7B* ATPase copper transporting beta, *CD45* cluster of differentiation 45, *CELF1* CUGBP Elav-like family member 1, *CFTR* cystic fibrosis transmembrane conductance regulator, *CLCN1* chloride voltage-gated channel 1, *COL4A5* collagen type IV alpha 5 chain, *DMPK* myotonic dystrophy protein kinase, *EAAT2* excitatory amino acid transporter 2, *EIF4E* eukaryotic translation initiation factor 4E, *FOXP3* forkhead box P3, *FOXP3fl* full-length FOXP3 isoform, *FOXP3Δ2* FOXP3 isoform lacking exon 2, *GH1* growth hormone 1, *HTT* huntingtin, *INSR* insulin receptor, *kDa* kilodalton, *LRRK2* leucine-rich repeat kinase 2, *MBNL1* muscleblind-like 1, *mRNA* messenger RNA, *NFAT5* nuclear factor of activated T cells 5, *pre-mRNA* precursor messenger RNA, *RBM20* RNA-binding motif protein 20, *RBM24* RNA-binding motif protein 24, *SF3B1* splicing factor 3b subunit 1, *SMN1* survival of motor neuron 1, *SMN2* survival of motor neuron 2, *snRNP* small nuclear ribonucleoprotein, *SRSF1* serine and arginine rich splicing factor 1, *TEX14* testis expressed 14, intercellular bridge forming factor, *TP53* tumor protein p53, *Treg* regulatory T cell, *TTN* titin, *VEGF* vascular endothelial growth factor

In the nervous system, splicing dysregulation not only triggers developmental arrest but also serves as a core driver of chronic neurodegenerative disorders. In Alzheimer’s disease (AD), aberrant aggregation of the SF U1 snRNP disrupts the splicing of key genes such as APP, thereby accelerating amyloid plaque deposition [153]. In Parkinson’s disease (PD), dysregulation of alternative splicing of genes including LRRK2 has been closely linked to the demise of dopaminergic neurons [154]. Studies on Huntington’s disease (HD) have revealed that mis-splicing of the HTT gene pre-mRNA generates highly cytotoxic truncated protein fragments, directly inducing neuronal damage and progressive neurodegeneration [155]. Furthermore, in multiple sclerosis (MS)—an autoimmune disorder—dysregulated expression of the SF CELF1 and its target gene NFAT5 has been observed in patient blood samples, implicating splicing dysregulation in the pathological progression of autoimmunity [156]. In amyotrophic lateral sclerosis (ALS), altered alternative splicing of EAAT2 mRNA reduces the expression of the glutamate transporter protein EAAT2, impairing glutamate reuptake and leading to excitotoxic neuronal death—a well-established key pathogenic mechanism in ALS [157].

Such splicing aberrations are equally prevalent in motor and circulatory system disorders. In spinal muscular atrophy (SMA), patients lack the essential SMN1 gene and rely on its paralog, SMN2. However, SMN2 frequently undergoes exon 7 skipping during splicing, producing an unstable and dysfunctional SMN protein, ultimately resulting in progressive skeletal muscle atrophy and motor neuron loss [170, 171]. In myotonic dystrophy, aberrant splicing also plays a well-established pathogenic role. Patients carry massive CTG repeat expansions in the DMPK gene; the resulting toxic RNA transcripts sequester splicing regulators such as MBNL1, disrupting the splicing of hundreds of downstream genes. Notably, mis-splicing of the chloride channel (CLCN1) directly accounts for the characteristic myotonia observed in these patients [172, 173]. In the circulatory system, RBM20 mutations lead to the retention of excessively compliant titin isoforms in the myocardium, directly impairing cardiac mechanical compliance and triggering dilated cardiomyopathy [158]. Additionally, the splicing regulator RBM24 has been implicated in heart failure progression by binding p53 mRNA and interacting with the translation initiation factor EIF4E, thereby blocking EIF4E binding and inhibiting translation initiation complex assembly [159, 180].

In the respiratory system, aberrant splicing constitutes a key mechanism underlying hereditary lung diseases and chronic obstructive pulmonary disease (COPD). For instance, splicing defects leading to loss of CFTR protein function cause mucociliary clearance dysfunction and cystic fibrosis [160–163]. In COPD, smoking-induced oxidative stress alters SFs such as SRSF1, increasing the ratio of the inhibitory VEGF165b isoform, which induces pulmonary microvascular endothelial cell apoptosis and compromises alveolar structure maintenance-a critical contributor to COPD progression [164].

In the hematopoietic and immune systems, mutations in core spliceosomal components (e.g., SF3B1) often precipitate transcriptomic catastrophe [165, 166]. By generating numerous aberrant transcripts harboring premature termination codons, these mutations block normal erythroid maturation and trigger myelodysplastic syndromes [167]. Concurrently, dysregulated splicing of immune receptors (e.g., CD45) or signaling molecules can disrupt immune homeostasis and precipitate autoimmune responses. For example, dysregulated splicing of the master transcription factor FOXP3 in Tregs, particularly an imbalance between the full-length isoform (FOXP3fl) and the exon-2-skipped variant (FOXP3Δ2), disrupts immune homeostasis by impairing Treg suppressive function, thereby precipitating autoimmune responses such as those observed in systemic lupus erythematosus and rheumatoid arthritis [168, 169].

Aberrant splicing has also been implicated in digestive system disorders. Wilson’s disease, an autosomal recessive disorder of copper metabolism primarily affecting the liver, is caused by defects in the ATP7B gene, which encodes a copper-transporting ATPase. Pathogenic mutations in ATP7B (e.g., c.2123 T > C) can create cryptic splice sites, misleading the spliceosome and resulting in mRNA transcripts with aberrant insertions or exon skipping. These defective mRNAs fail to translate into functional ATP7B protein, impairing copper excretion into bile and leading to progressive hepatic and neurological copper accumulation, cirrhosis, and neuropsychiatric symptoms [174, 175].

In the endocrine system, splicing abnormalities often underpin hormone synthesis defects and receptor desensitization. For example, mis-splicing of insulin receptor (INSR) exon 11 reduces its ligand affinity, serving as a molecular basis for certain forms of refractory diabetes [176, 181]. Similarly, aberrant skipping of GH1 gene exon 3 generates a 17.5 kDa growth hormone variant lacking normal activity; this isoform exerts a dominant-negative effect, impairing secretion of functional growth hormone and ultimately leading to somatotroph cell death in the pituitary—a key etiology of isolated growth hormone deficiency type II (IGHD II) [177].

In the reproductive and urinary systems, aberrant splicing has emerged as a significant pathogenic factor. In azoospermia, loss-of-function variants in TEX14, including splice-site-disrupting variants predicted to impair donor-site recognition, can reduce TEX14 function, disrupt intercellular bridge formation during meiosis, and lead to meiotic arrest and spermatogenic failure [178]. In the urinary system, Alport syndrome pathogenesis involves splice-site mutations in COL4A5, with studies indicating that approximately 10–15% of X-linked Alport syndrome cases arise from such mutations [179].

In summary, aberrant RNA splicing represents a core pathogenic mechanism spanning multiple organ systems. Through exon skipping, cryptic exon activation, or isoform imbalance, it drives disease by causing loss of critical protein function or aberrant activation of signaling pathways.

## Therapeutic strategies targeting RNA splicing

RNA splicing dysregulation is a central pathogenic driver of numerous human diseases, spanning cancer, neurological disorders, cardiovascular and metabolic diseases, and inherited conditions. Consequently, targeting the splicing machinery and its regulatory networks has emerged as a promising therapeutic paradigm, with strategies ranging from small-molecule modulators to antisense oligonucleotides to novel gene-editing technologies. This section summarizes the key therapeutic approaches targeting RNA splicing, highlighting their mechanisms of action, representative disease applications, clinical progress, and translational potential (Table 2).

**Table 2:** Therapeutic strategies targeting RNA splicing: molecular targets, mechanisms and clinical applications

Primary Category	Subtype	Core Targets	Brief Mechanism of Action	Representative Drugs & Technologies	Indications
**Small-molecule modulators targeting core spliceosome components**	Spliceosome inhibitors/disruptors	SF3B1, U4/U5/U6 tri-snRNP	Disrupt spliceosome assembly, induce intron retention and nonsense-mediated decay, and exploit spliceosome dependence in cancer cells	Pladienolide B, H3B-8800, FR901464, Sudemycin E, FD-895, Herboxdiene, Isoginkgetin	Hematological malignancies, fibrosarcoma, melanoma, SMA
	Splicing enhancers	Interface between SMN2 pre-mRNA and U1 snRNP	Stabilize spliceosomal recognition of a weak exon and promote productive exon inclusion	Risdiplam	SMA
	Allosteric modulators	RBM20, IKBKAP	Bind allosteric sites of splicing factors to alter protein conformation, and selectively modulate RNA recognition and splicing of specific genes	TTN splicing-targeted modulators, Kinetin	Dilated cardiomyopathy, familial dysautonomia
**Small-molecule inhibitors against post-translational modification enzymes of splicing factors**	Splicing factor phosphokinase inhibitors	CLKs, SRPKs, DYRKs	Inhibit phosphorylation of SR proteins and other splicing factors, remodel the alternative splicing landscape	SM08502, Alectinib, SPHINX, SPHINX31, LDN192960, Leucettinib-21	Colorectal cancer, prostate cancer, TNBC, MM, wAMD, diabetic retinopathy, Alzheimer’s disease, Down syndrome
	Other modifying enzyme inhibitors	HDACs, PRMTs	Regulate acetylation and methylation of splicing factors to fine-tune abnormal splicing patterns, with milder side effects than broad-spectrum spliceosome inhibitors	Romidepsin, GSK3326595	Multiple solid tumors and hematological malignancies, spinal muscular atrophy, hereditary cardiomyopathy, chronic inflammatory diseases
**Antisense oligonucleotide therapy**	RNase H1-mediated degradative ASOs	SOD1, APOC3, KLKB1, HBV RNAs, DGAT2, FANCM, FOXP3	DNA-containing gapmer ASOs hybridize with target transcripts and recruit RNase H1, resulting in target-RNA cleavage and reduced expression of the encoded proteins	Tofersen, olezarsen, donidalorsen, bepirovirsen, ION224, FANCM-targeting ASOs, AZD8701	ALS, FCS, HAE, CHB, MASH, ALT-positive cancers and advanced solid tumors
	Steric-blocking splice-modulating ASOs	SMN2, SCN1A, TRA2B	ASOs bind splice sites or cis-regulatory elements without recruiting RNase H1, thereby altering spliceosome or splicing-factor recognition and promoting productive exon inclusion, poison-exon exclusion or poison-exon inclusion	Nusinersen, zorevunersen, ASO-1570	SMA, Dravet syndrome, and preclinical GBM and TNBC
**Other novel therapeutic approaches targeting RNA splicing**	Immunotherapy mediated by neoantigens derived from alternative splicing	Tumor-specific alternative splicing loci	Screen neoantigens uniquely generated by tumor-specific splicing variations to construct personalized cancer vaccines or TCR-T cell therapies	Individual antigenic peptides screened by multi-omics combined with bioinformatic analysis	Various malignant solid tumors and hematological malignancies
	Trans-splicing RNA exon editing	Aberrant exons on defective pre-mRNA	Deliver RNA vectors carrying correct sequences to replace defective exons via endogenous cellular spliceosomes, enabling the repair of oversized disease-causing genes	ACDN-01	Stargardt disease
	CRISPR gene/RNA editing	RBM20	The SCISSOR system supports cleavage of RNA fragments of arbitrary lengths. Cas9-mediated genome editing permanently modifies splicing-regulatory genes to restore normal splicing	Type III CRISPR-Csm SCISSOR system, RBM20 site-specific editing tools	Tay-Sachs disease, cardiomyopathy induced by heart failure, combined immunotherapy for tumors

*Abbreviations*: *ALS* amyotrophic lateral sclerosis, *ALT* alternative lengthening of telomeres, *APOC3* apolipoprotein C3, *ASO* antisense oligonucleotide, *Cas9* CRISPR-associated protein 9, *CHB* chronic hepatitis B, *CLK* CDC-like kinase, *CRISPR* clustered regularly interspaced short palindromic repeats, *Csm* type III-A CRISPR-Cas interference complex, *DGAT2* diacylglycerol O-acyltransferase 2, *DYRK* dual-specificity tyrosine-phosphorylation-regulated kinase, *FANCM* FA complementation group M, *FCS* familial chylomicronemia syndrome, *FOXP3* forkhead box P3, *GBM* glioblastoma, *HAE* hereditary angioedema, *HBV* hepatitis B virus, *HDAC* histone deacetylase, *IKBKAP* inhibitor of nuclear factor kappa B kinase complex-associated protein (current gene symbol: ELP1), *KLKB1* kallikrein B1, *MASH* metabolic dysfunction-associated steatohepatitis, *MM* multiple myeloma, *pre-mRNA* precursor messenger RNA, *PRMT* protein arginine methyltransferase, *RBM20* RNA-binding motif protein 20, *RNA* ribonucleic acid, *RNase H1* ribonuclease H1, *SCISSOR* selective cleavages and intramolecular stitches of RNA, *SCN1A* sodium voltage-gated channel alpha subunit 1, *SF3B1* splicing factor 3b subunit 1, *SMA* spinal muscular atrophy, *SMN2* survival of motor neuron 2, *snRNP* small nuclear ribonucleoprotein, *SOD1* superoxide dismutase 1, *SR* protein, serine/arginine-rich protein, *SRPK* serine/arginine-rich protein-specific kinase, *TCR-T* T-cell receptor-engineered T-cell therapy, *TNBC* triple-negative breast cancer, *TRA2B* transformer 2 beta homolog, *TTN* titin, *U1 snRNP* U1 small nuclear ribonucleoprotein, *U4/U6.U5 tri-snRNP* U4/U6.U5 tri-small nuclear ribonucleoprotein complex, *wAMD* wet age-related macular degeneration

### Small-molecule modulators targeting core spliceosomal components

**Fig. 4:**
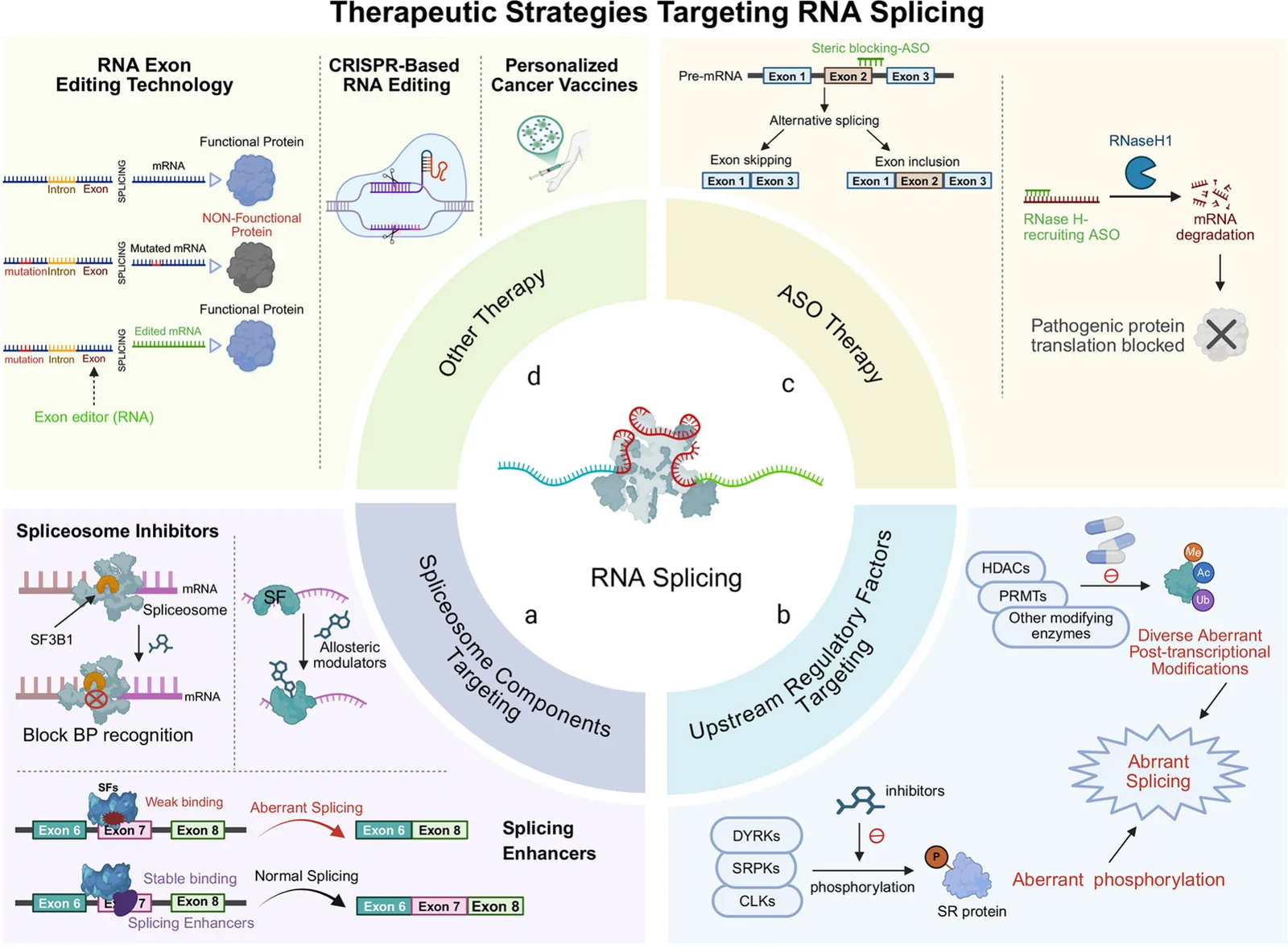
Therapeutic strategies targeting RNA splicing. **a** Small-molecule modulators targeting core spliceosomal components. Spliceosome inhibitors competitively occupy the branch point recognition pocket on SF3B1, preventing proper spliceosome assembly and disrupting splicing; Under pathological conditions, the interaction between SFs and pre-mRNA is weak, making the transcript prone to abnormalities such as exon skipping. This class of drugs stabilizes the interaction between SFs and pre-mRNA, thereby maintaining canonical splicing; Allosteric modulators induce conformational changes in SFs upon binding to the allosteric site. This structural reorganization ultimately influences the factor’s recognition and binding affinity for specific RNA sequences, leading to regulated splicing. **b** Small-molecule inhibitors targeting key splicing factor kinases. Targeting phosphorylation enzymes; Targeting other post-translational modification enzymes. **c** ASO drugs primarily function through two mechanisms: Induction of RNA degradation: After RNase H-recruiting ASO drugs bind to RNA, endogenous RNase H1 recognizes and cleaves the RNA strand, leading to degradation of the target transcript and subsequent downregulation of pathogenic protein expression; Modulation of splicing via steric hindrance; After steric block ASO drugs bind to specific mRNA sequences, it prevents spliceosome recognition, thereby regulating the splicing process. **d** Other therapeutic strategies targeting RNA splicing. Trans-splicing RNA exon editing technology; Novel CRISPR-based RNA editing tools; Designing personalized therapeutic cancer vaccines utilizing neoantigens derived from alternative splicing. Abbreviations: Ac, acetylation; ASO, antisense oligonucleotide; BP, branch point; CLKs, CDC-like kinases; CRISPR, clustered regularly interspaced short palindromic repeats; DYRKs, dual-specificity tyrosine-phosphorylation-regulated kinases; HDACs, histone deacetylases; Me, methylation; mRNA, messenger RNA; P, phosphorylation; PRMTs, protein arginine methyltransferases; RNA, ribonucleic acid; RNase H1, ribonuclease H1; SF, splicing factor; SF3B1, splicing factor 3b subunit 1; SFs, splicing factors; SR protein, serine/arginine-rich protein; SRPKs, serine/arginine-rich protein-specific kinases; Ub, ubiquitination

Direct targeting of core spliceosome components represents a pivotal therapeutic strategy for intervening in aberrant splicing. These drugs are mostly derived from natural products. They work by binding to key subunits of the spliceosome, causing conformational changes that affect the assembly and catalytic activity of the splicing complex. Based on their mechanisms of action, these drugs can be broadly divided into three categories (Fig. 4a).

#### Spliceosome inhibitors/disruptors

These drugs broadly disrupt RNA splicing by interfering with one or more steps in spliceosome assembly. They are primarily used in oncology, with the most extensively studied targeting SF3B1, a core component of the U2 snRNP [182]. Represented by the macrolide compound pladienolide B and its oral derivative H3B-8800, these molecules competitively occupy the branch point recognition pocket of SF3B1, thereby blocking early spliceosome assembly on pre-mRNA [182–184]. In hematologic malignancies carrying SF3B1 or U2AF1 mutations, these modulators exhibit a pronounced "synthetic lethal" effect: they induce widespread IR, leading to transcripts with premature termination codons that trigger nonsense-mediated mRNA decay, resulting in selective killing of spliceosome-dependent cancer cells [183, 185]. Additional natural products and derivatives targeting SF3B1 have also been identified, including FR901464, Sudemycin E, FD-895, and Herboxdiene [185–188]. Furthermore, the natural biflavonoid Isoginkgetin represents another classic splicing inhibitor, blocking recruitment of the U4/U6.U5 tri-snRNP complex, and thereby preventing the transition of the spliceosome from complex A to complex B and disrupting splicing progression. In fibrosarcoma and melanoma models, Isoginkgetin has been shown to effectively inhibit cell invasion and induce apoptosis, contributing to its anticancer effects [189].

Beyond anti-cancer applications, research on spliceosome inhibitors has been increasingly extended to non-neoplastic diseases in recent years, opening up brand-new avenues for their clinical application. For instance, studies have verified that low-dose SF3B1 inhibitors are capable of regulating the splicing of SMN2 pre-mRNA, markedly increasing the inclusion level of exon 7 in SMN2 and producing full-length SMN protein with physiological activity. In patient-derived cellular models of SMA, this intervention ameliorates pathological phenotypes associated with motor neuron damage, which firmly validates the therapeutic potential of SF3B1 inhibitors against spinal muscular atrophy [190].

#### Splicing enhancers

In contrast to spliceosome inhibitors, this class of agents promotes the inclusion of specific exons by stabilizing weak interactions between SFs and pre-mRNA, thereby restoring functional protein expression. This strategy has shown unique therapeutic value in the treatment of genetic disorders. Risdiplam is a representative example of this class. It is used in the treatment of SMA. In SMA pathogenesis, patients lack a functional SMN1 gene, while the paralogous SMN2 gene contains a critical nucleotide transition that causes exon 7 to be frequently skipped by the spliceosome, resulting mainly in an unstable and defective protein. Acting as a molecular glue, Risdiplam precisely binds to the interface between SMN2 pre-mRNA and U1 snRNP, efficiently promoting exon 7 inclusion, thereby correcting the splicing defect and increasing the production of functional SMN protein. This conceptual shift from "toxic killing" to "functional enhancement" provides an important paradigm for developing targeted therapies for other rare and chronic diseases caused by splicing defects [191].

#### Allosteric modulators

Unlike direct inhibition or enhancement, allosteric modulators bind to non-active sites (allosteric sites) on SFs, inducing conformational changes that subsequently influence the recognition of specific RNA sequences. With advances in structural biology, these drugs have shown significant potential for clinical application in non-neoplastic diseases. For example, RBM20 is a key protein that regulates myocardial elasticity. It determines the splicing pattern of *TTN*—specifically, whether the elastic short isoform (N2B) or the less elastic long isoform (N2BA) is produced. In dilated cardiomyopathy, mutations or loss of RBM20 lead to pathological splicing of the *TTN* gene, resulting in an overabundance of the long, inelastic N2BA isoform. Researchers have identified allosteric modulators that regulate spliceosome recognition of exons within the PEVK region of *TTN*, biasing splicing toward the more elastic N2B isoform and restoring myocardial elasticity [192]. Additionally, Kinetin has been shown to correct IKBKAP exon 20 missplicing in familial dysautonomia (FD) [193]. These advances highlight the unique therapeutic promise of allosteric spliceosome modulators in genetic, cardiovascular, and chronic diseases, positioning them as an important direction in the field of RNA-targeted therapies.

### Small-molecule inhibitors targeting key splicing factor kinases

Aberrant splicing can also be targeted indirectly through enzymes that regulate SF post-translational modifications. By altering SF localization, RNA binding, and spliceosome interactions, inhibitors of SF kinases and related enzymes can remodel disease-associated splicing with potentially greater selectivity than broad spliceosome inhibitors (Fig. 4b).

#### Drugs targeting key splicing factor phosphorylation enzymes

The precision of the splicing process is highly dependent on the phosphorylation status of SFs (e.g., SR proteins), which is tightly regulated by various splicing kinases, including Cdc2-like kinases (CLKs), SR protein-specific kinases (SRPKs), and dual-specificity tyrosine-phosphorylation-regulated kinases (DYRKs). Small-molecule inhibitors targeting these kinases systematically remodel the splicing landscape of cells, offering a targeted approach distinct from direct spliceosome inhibition [194, 195]. Such drugs have broad prospects in research and clinical application for two major categories of diseases: malignant tumors, as well as non-neoplastic chronic, hereditary, and degenerative disorders.

In the treatment of malignant tumors, these inhibitors exert anti-tumor effects through multiple dimensions, including inhibiting cell proliferation, anti-angiogenesis, reversing drug resistance, and blocking oncogenic signaling pathways. For instance, the CLK inhibitor SM08502 reduces SR protein phosphorylation to inhibit the constitutive splicing of Wnt signaling pathway genes, thereby demonstrating potent anti-proliferative activity in solid tumor models such as colorectal cancer. Alectinib, a clinically approved ALK inhibitor, has been found in recent studies to have strong SRPK inhibitory activity and thus achieve broad-spectrum anti-tumor effects [196].

SRPK1 inhibitors, including SPHINX and its optimized analog SPHINX31, also exhibit prominent clinical application potential in the field of tumor therapy [197]. In prostate cancer, SRPK1 inhibition modulates the alternative splicing of VEGF, shifting the splicing pattern from pro-angiogenic to anti-angiogenic isoforms, which effectively suppresses tumor blood supply and growth [197]. Due to their ability to reduce choroidal neovascularization (CNV), SPHINX and SPHINX31 are currently being explored for the treatment of wet age-related macular degeneration (wAMD) and diabetic retinopathy [198, 199]. Furthermore, research into small-molecule inhibitors targeting DYRKs is actively progressing. Studies indicate that LDN192960, a specific DYRK2 inhibitor, can mitigate the progression of triple-negative breast cancer and multiple myeloma by suppressing DYRK2 activity and subsequently reducing proteasome activity [200].

In the field of non-neoplastic diseases, these drugs are mainly applied to ocular angioproliferative lesions, neurodegenerative diseases, and hereditary encephalopathies. For wet age-related macular degeneration and diabetic retinopathy, SRPK1 inhibitors SPHINX and SPHINX31 are capable of inhibiting the formation of abnormal choroidal neovascularization and alleviating fundus exudation and tissue damage, and have currently entered the preclinical research phase for ophthalmic disorders [201, 202]. Additionally, the DYRK1A inhibitor Leucettinib-21 is being developed as a clinical candidate for Alzheimer’s disease and Down syndrome [200]. Unlike inhibitors that directly target the spliceosome, kinase inhibitors only regulate splicing preference instead of completely blocking splicing reactions. Accordingly, they possess a wider therapeutic index in clinical application and serve as an indispensable component within the system of post-transcriptional RNA-targeted therapy [203].

#### Drugs targeting other post-translational modification enzymes

In addition to phosphorylation, modifications such as acetylation and methylation of SFs also influence splicing processes. Targeted drugs developed against these post-translational modification enzymes also have a broad spectrum of disease indications and exhibit prominent therapeutic potential in malignant tumors as well as a variety of non-neoplastic diseases. In anti-tumor therapy, the histone deacetylase inhibitor romidepsin can interfere with the aberrant splicing of the BRCA1 gene and restore the normal expression of transcripts related to this tumor suppressor gene, so as to correct the disordered DNA damage repair in tumor cells [204]. The protein arginine methyltransferase inhibitor GSK3326595 induces alternative splicing of MDM4, relieves its inhibitory effect on the p53 pathway, reactivates tumor-suppressive signals in tumor cells, and effectively halts the progression of a variety of solid tumors and hematological malignancies [205]. At present, multiple small-molecule compounds targeting enzymes responsible for acetylation and methylation modification of SFs have entered preclinical research for oncology indications [206, 207].

In the field of non-neoplastic diseases, drugs that regulate splicing via acetylation and methylation modifications can be applied to treat hereditary diseases, chronic inflammatory disorders, and other illnesses. Instead of completely blocking the fundamental splicing function of cells, these targeted drugs only fine-tune splicing patterns to rectify abnormal transcripts under pathological conditions, thus featuring more manageable side effects compared with broad-spectrum splicing inhibitors [208, 209]. For example, HDAC inhibitors are able to correct the abnormal splicing of the SMN2 gene in spinal muscular atrophy, increase the production of functional SMN protein, and slow the injury of motor neurons, making them potential candidates for the treatment of neurodegenerative diseases. Small-molecule drugs targeting acetylation-modifying enzymes can fix the aberrant splicing of disease-causing genes in cardiomyopathy, improve the structure and contractile function of cardiomyocytes, and alleviate the progression of hereditary cardiomyopathy [210, 211].

### Splice-switching antisense oligonucleotide therapy

Antisense oligonucleotides (ASOs) are a class of oligonucleotides capable of inducing gene silencing. Typically, ASOs consist of 12–30 nucleotides and are designed to be antisense-complementary to the target RNA [212]. Through complementary base pairing, ASOs precisely bind to specific sequences of the target pre-mRNA. Their primary mechanism of action involves steric hindrance, which interferes with the recognition of splice sites by the splicing machinery, thereby enabling the “programming” of the splicing process and the precise regulation of gene expression at the RNA level [213]. In recent years, breakthroughs in chemical modifications and delivery technologies have significantly accelerated the development and approval of ASO drugs, bringing new therapeutic hope for numerous malignancies, genetic disorders, and even rare diseases [213].

In the field of oncology, the core strategies primarily involve two mechanisms: induction of target RNA degradation and modulation of splicing (Fig. 4c) [214]. FANCM-targeting ASOs represent a degradative ASO approach for ALT-positive cancers, in which RNase H1-mediated depletion of FANCM transcripts induces telomeric dysfunction and suppresses tumor growth in preclinical models [215]. Similarly, AZD8701 targets FOXP3 mRNA to reduce regulatory T-cell-associated immunosuppressive programmes and has been evaluated in patients with advanced solid tumors [216]. In parallel, steric-blocking splice-modulating ASOs provide a non-degradative strategy for reshaping oncogenic splicing programmes. ASO-1570 targets TRA2B regulatory elements to promote poison-exon inclusion, reduce TRA2B protein expression and impair tumor-cell growth, with preclinical activity reported in glioblastoma and triple-negative breast cancer models [217].

In non-malignant disorders, ASO drugs similarly exert therapeutic effects through the aforementioned two mechanisms, achieving breakthrough progress in genetic disorders, neurodegenerative diseases, cardiovascular and metabolic diseases, and infectious diseases. In terms of splicing modulation, nusinersen promotes the inclusion of exon 7 in the SMN2 gene, significantly increasing functional SMN protein expression and fundamentally transforming the treatment landscape for spinal muscular atrophy [218, 219]. Moreover, zorevunersen has shown therapeutic potential in Dravet syndrome [220]. RNase H1-mediated degradative ASOs instead reduce pathogenic RNA or protein expression. Olezarsen targeting APOC3 has been approved in the EU for familial chylomicronemia syndrome, while ION224 targeting DGAT2 has shown positive results in Phase II clinical trials for metabolic dysfunction-associated steatohepatitis [221]. Donidalorsen was recently approved by the FDA for hereditary angioedema, whereas the hepatitis B virus-targeting bepirovirsen is currently under clinical evaluation for chronic hepatitis B [222, 223].

### Other therapeutic strategies targeting RNA splicing

Beyond small-molecule inhibitors targeting core spliceosome components and their kinases, as well as splice-switching ASOs, a variety of novel therapeutic strategies have emerged in recent years, expanding the boundaries of RNA splicing-targeted interventions (Fig. 4d).

In the field of immunotherapy, the design of personalized therapeutic cancer vaccines utilizing neoantigens derived from alternative splicing represents an emerging direction. Aberrant alternative splicing generates neoantigens that are not expressed or are minimally expressed in normal cells. By integrating omics sequencing with bioinformatic analysis of tumor-specific alternative splicing events, immunogenic peptides with high affinity for major histocompatibility complex (MHC) molecules can be identified, providing candidate antigens for personalized immunotherapies such as therapeutic cancer vaccines and T-cell receptor-engineered T cells (TCR-T cells) [224, 225].

At the level of gene repair, trans-splicing RNA exon editing is emerging as one of the most clinically promising directions in the field of RNA splicing targeting. This technology employs engineered precursor molecules carrying correct RNA sequences and splicing signals, leveraging the endogenous spliceosome to replace defective exons on pre-mRNA with functional ones. Its core advantages include the ability to repair large genes (e.g., ABCA4) that are challenging for conventional gene therapy, enabling “one drug for multiple diseases “ by addressing various mutations within the same exon, and offering high safety due to its reversible nature and the absence of exogenous enzyme introduction [226, 227]. In 2024, ACDN-01, developed by Ascidian Therapeutics, received FDA approval to enter clinical trials for the treatment of Stargardt disease, becoming the first investigational drug in this modality globally to advance to the clinical stage, marking the formal transition of RNA exon editing from laboratory research to clinical validation [26].

At the level of gene editing, novel CRISPR-based RNA editing tools have also achieved precise regulation of RNA splicing. The SCISSOR technology, developed by Sun et al., utilizes an engineered type III CRISPR-Csm complex to achieve flexible deletion of RNA fragments of arbitrary length. This breakthrough not only provides a novel strategy for correcting genetic disorders caused by frameshift mutations (e.g., Tay-Sachs disease) but also enables the artificial induction of frameshift mutations in tumor cells to generate neoantigens, opening new avenues for combination strategies in cancer immunotherapy [228]. Additionally, CRISPR/Cas9-mediated editing of RBM20 restores normal RNA splicing in cardiomyocytes and improves cardiac function, significantly enhancing survival in preclinical heart failure models [229].

## Conclusions and perspectives

RNA splicing is a central and multifaceted layer of eukaryotic post-transcriptional gene regulation. Through its dynamic molecular machinery, it expands proteomic diversity, directs cellular identity, and coordinates organismal development, homeostasis, and stress responses. In this review, we systematically elucidate the molecular mechanisms of RNA splicing and the roles of epigenetic factors—including DNA methylation, m6A and m7G RNA modifications, RNA editing, ncRNAs, and histone modifications—in the splicing process. We further explore how alternative splicing participates in physiological processes such as growth, development, and tissue-specific physiological functions, and how the disruption of splicing homeostasis leads to cancer and diverse non-neoplastic diseases across organ systems.

With the deepening of research into RNA splicing mechanisms, targeting RNA splicing has become a prominent entry point for drug development. Several classes of splicing‑targeted therapeutics have matured, with applications spanning inflammatory, degenerative and malignant disorders. Innovative drugs have entered the clinical validation stage: for example, small-molecule modulators targeting core spliceosomal components (e.g., H3B-8800, risdiplam), kinase inhibitors targeting the phosphorylation of SFs (e.g., SM08502, SPHINX31), and ASOs that regulate splicing (e.g., nusinersen, eteplirsen). These drugs have demonstrated transformative efficacy in oncology, rare genetic diseases, and chronic conditions. Furthermore, emerging therapeutic modalities, such as RNA exon editing (represented by ACDN-01) and CRISPR-based RNA editing tools (e.g., SCISSOR), are further expanding the therapeutic frontier, offering new possibilities for repairing large gene defects and achieving the goal of "one drug covering multiple mutations."

Despite these remarkable advances, the field of splicing research and therapeutic translation still faces several fundamental challenges and unmet needs. At the mechanistic level, the “splicing code” has not been fully deciphered. First, robust computational frameworks capable of accurately predicting the impact of single mutations or environmental perturbations on global splicing are lacking. Second, research on the structural dynamics of early splicing complexes remains insufficient. Currently resolved spliceosome structures are mostly static snapshots of specific functional states, whereas the spliceosome is inherently a highly dynamic molecular machine. The transient conformational changes, intermediate states and transition‑state structures that occur during catalysis remain difficult to capture. Deducing dynamic behavior from static structures and revealing the temporal patterns of coordinated movement among various components remains a primary research hurdle. Additionally, the spatiotemporal distribution of SFs within phase-separated condensates and their pathological remodeling under cellular stress require further exploration. At the translational level, the specificity and safety of drugs targeting RNA splicing constitute the most significant bottlenecks. Regarding specificity, the difficulty for small-molecule modulators lies in targeting aberrant splicing while avoiding interference with essential normal splicing events. For instance, although H3B-8800 (targeting SF3B1) can selectively kill tumor cells harboring SF mutations via synthetic lethality, off-target risks remain, which may induce myelosuppression or adverse cardiovascular events. The challenge for ASO drugs lies in their lack of tissue specificity in vivo and the risk of activating immune responses. RNA editing tools primarily face difficulties regarding off-target editing and the evaluation of long-term genetic effects. Safety issues permeate every stage of clinical translation. Because small-molecule modulators struggle to distinguish between mutant and wild-type spliceosomes, they often lead to systemic toxicities such as gastrointestinal reactions, myelosuppression, and cardiovascular toxicity. ASOs face immunogenicity risks and accumulation in non-target tissues, manifesting as thrombocytopenia, hepatorenal impairment, and inflammation at the injection site. Furthermore, as tissue sensitivity to splicing perturbations varies significantly, rapidly proliferating tissues are easily damaged, while non-proliferative tissues like the myocardium and neurons may also suffer irreversible injury. In combination therapies, splicing modulators may further affect the metabolic and toxicological profiles of other drugs.

Looking ahead, the evolution of splicing research and therapy will advance along the following trajectories. First, the fusion of multi-omics analysis and artificial intelligence will facilitate the deciphering of the splicing code. Combining large-scale cancer genomic, transcriptomic, and proteomic datasets with deep learning architectures (such as next-generation SpliceAI models) will enable end-to-end prediction from DNA sequence to splicing outcome, promoting the systematic identification of pathogenic splicing variants and druggable targets. Second, the diversification and precision of targeted interventions will accelerate clinical translation. With advances in delivery technologies, such as stereopure oligonucleotides, antibody-oligonucleotide conjugates (AOCs), and lipid nanoparticles (LNPs), the tissue specificity of splicing interventions will be significantly enhanced. Simultaneously, “controllable switches” developed via small-molecule induction or optogenetics will further improve spatiotemporal precision. Third, the integration of splicing medicine and immunotherapy will provide new solutions for cancer treatment. Personalized vaccines and TCR-T cell therapies targeting tumor-specific splicing-derived neoantigens, along with strategies to remodel the tumor immune microenvironment through RNA editing-induced immunogenicity, are advancing from preclinical validation toward early clinical trials. Fourth, the expansion from rare diseases to common diseases will become the primary direction for targeted splicing therapies. Following the successful paradigm of risdiplam for spinal muscular atrophy, drug development targeting splicing is increasingly penetrating major chronic disease areas, including Alzheimer’s disease, heart failure, and metabolic syndrome [16, 151, 153, 230].

In conclusion, RNA splicing research is undergoing a fundamental shift from mechanistic discovery to mechanism-driven therapeutic translation. The interdisciplinary fusion of basic biology with computational analysis and materials science is reshaping the landscape of splicing medicine. In the coming decade, as our understanding of splicing mechanisms deepens, targeted splicing therapies will accelerate their transition from the laboratory to the clinic, ultimately transforming splicing dysregulation from a disease driver into a tractable target for clinical intervention.

## Data Availability

Not applicable.
